# Neoadjuvant therapy alters the immune microenvironment in pancreatic cancer

**DOI:** 10.3389/fimmu.2022.956984

**Published:** 2022-09-26

**Authors:** Huiru Zhang, Longyun Ye, Xianjun Yu, Kaizhou Jin, Weiding Wu

**Affiliations:** ^1^ Department of Pancreatic Surgery, Shanghai Cancer Centre, Fudan University, Shanghai, China; ^2^ Department of Oncology, Shanghai Medical College, Fudan University, Shanghai, China; ^3^ Shanghai Pancreatic Cancer Institute, Fudan University, Shanghai, China; ^4^ Pancreatic Cancer Institute, Fudan University, Shanghai, China

**Keywords:** pancreatic cancer, tumor environment (TME), immune, immunology, neoadjuvant therapy

## Abstract

Pancreatic cancer has an exclusive inhibitory tumor microenvironment characterized by a dense mechanical barrier, profound infiltration of immunosuppressive cells, and a lack of penetration of effector T cells, which constitute an important cause for recurrence and metastasis, resistance to chemotherapy, and insensitivity to immunotherapy. Neoadjuvant therapy has been widely used in clinical practice due to its many benefits, including the ability to improve the R0 resection rate, eliminate tumor cell micrometastases, and identify highly malignant tumors that may not benefit from surgery. In this review, we summarize multiple aspects of the effect of neoadjuvant therapy on the immune microenvironment of pancreatic cancer, discuss possible mechanisms by which these changes occur, and generalize the theoretical basis of neoadjuvant chemoradiotherapy combined with immunotherapy, providing support for the development of more effective combination therapeutic strategies to induce potent immune responses to tumors.

## 1 Introduction

Pancreatic cancer, which is highly malignant and difficult to treat, is ranked fourth among cancer-related deaths in the United States and is anticipated to be the second highest contributor to cancer death by 2030 (1, 2). Surgery is the only potential cure for pancreatic cancer. However, due to the high primary irresectability rate and many postoperative complications, the 5-year survival rate is less than 30% in patients who undergo surgery (3). Adjuvant therapy increases survival benefits to some extent but may cause bone marrow suppression and immune deficiency (4). In addition, a proportion of patients fail to receive the designed adjuvant therapy because of postoperative complications, delayed surgical recovery, early disease recurrence, and other reasons (5, 6). Neoadjuvant therapy is considered to be a promising therapeutic strategy, which is mainly reflected in the following: a) it can transform the initial marginal or locally unresectable tumors into resectable tumors and increase the number of surgical candidates, b) it can improve the R0 resection rate and overall survival, c) it can eliminate circulating tumor cells and distant micrometastases as well as reduce postoperative recurrence, and d) it can identify patients with progressive disease unsuitable for surgical intervention (7–11). First-line neoadjuvant therapies include a combination chemotherapy regimens consisting of oxaliplatin, irinotecan, fluorouracil, and leucovorin (FOLFIRINOX) or gemcitabine combined with nab-paclitaxel (AG), and many clinical trials have reported their beneficial effects on pancreatic cancer (12–14).

Cancer development is the result of complex and dynamic interactions between tumor cells and the tumor microenvironment (TME). The TME is a highly complex system consisting of tumor cells, immune cells, stromal cells, and non-cellular components, including extracellular matrix and soluble products, such as chemokines and cytokines (15). Pancreatic cancer has an exclusive inhibitory TME characterized by a dense physical barrier and the profound infiltration of immunosuppressive cells (16). Malignant epithelial cells account for only approximately 20% of the tumor bulk, while the desmoplastic stroma formed by cancer-associated fibroblasts (CAFs), collagens, and hyaluronic acid accounts for approximately 80% of the tumor mass (17). In addition, a large number of myeloid-derived suppressor cells (MDSCs), tumor-associated macrophages (TAMs), and regulatory T cells (Tregs), as well as limited effector T cells, may contribute to the immunosuppressive characteristics of the TME (18). These components constitute important causes of pancreatic cancer recurrence, metastasis, resistance to chemotherapy, and insensitivity to immunotherapy (16). Therefore, understanding how neoadjuvant therapy affects stromal components and the antitumor immune response will help to develop new combined immunotherapy strategies for treating this lethal cancer in the future.

**Figure 1:**
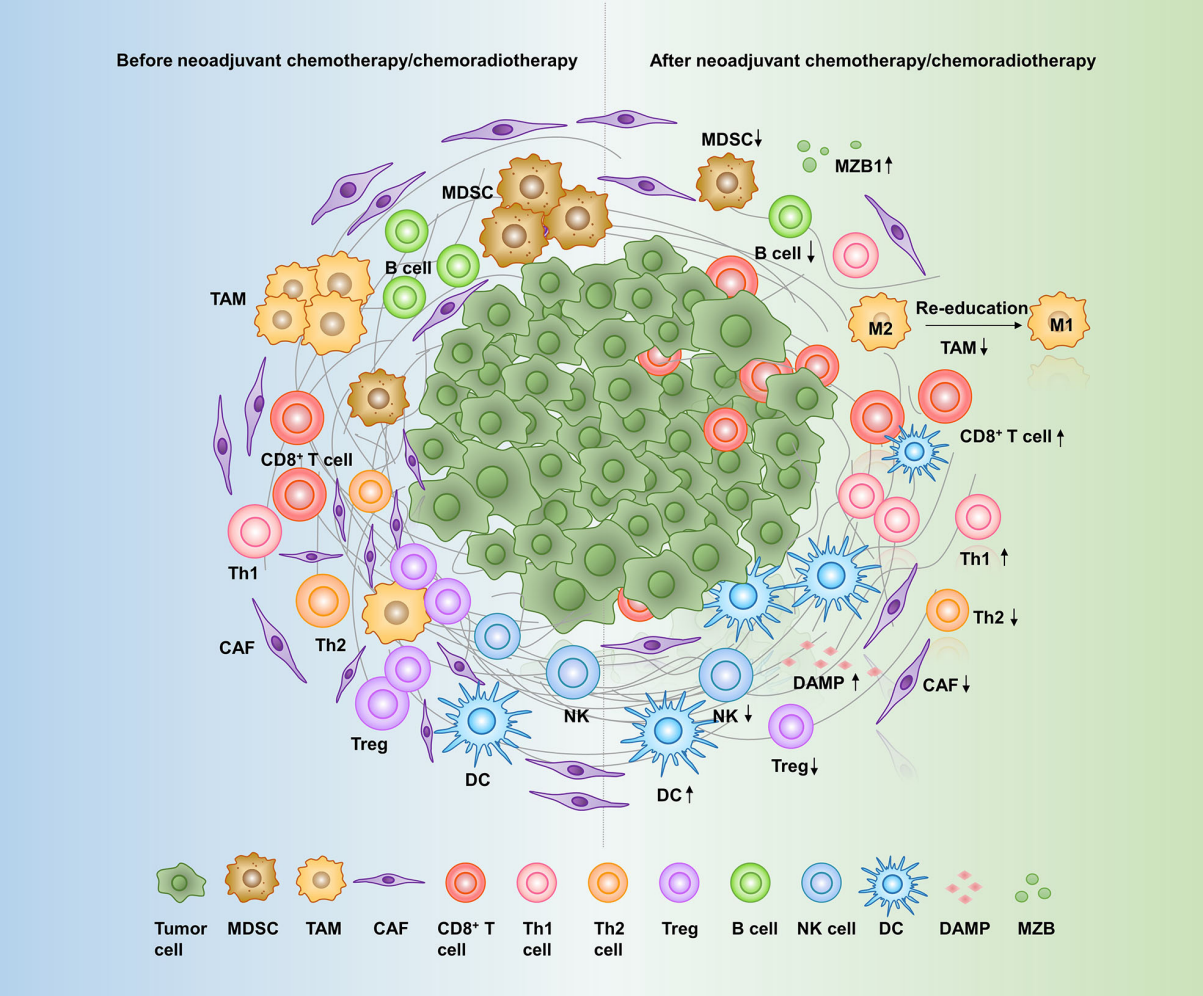
Effects of neoadjuvant therapy on the pancreatic cancer microenvironment. A variety of cells and molecules in the pancreatic cancer microenvironment are altered in response to neoadjuvant therapy: CD8^+^ T cells, Th1 cells, DCs, DAMPs, and MZB1 are increased, while CAFs, Th2 cells, Tregs, MDSCs, TAMs, NK cells, and B cells are decreased. MDSC, myeloid-derived suppressor cell; TAM, tumor-associated macrophage; CAF, cancer-associated fibroblast; Th cell, T helper cell; Treg, regulatory T cell; NK cell, natural killer cell; DC, dendritic cell; DAMPs, damage-associated molecular patterns; MZB1, Marginal zone B- and B1-cell-specific protein.

This review concludes several aspects of neoadjuvant therapy-induced alterations in the pancreatic cancer microenvironment (**Figure 1**) and discusses the possible mechanisms of these changes, providing a basis for the development of more promising combination therapies with immunotherapy in a neoadjuvant setting. The methods for reference screening are summarized in **Supplementary Figure 1**.

## 2 Effects of neoadjuvant therapy on the pancreatic tumor microenvironment

### 2.1 Cancer-associated fibroblasts and extracellular matrix

CAFs are the major cellular components of the pancreatic cancer stroma and can secrete cytokines, growth factors, and extracellular matrix (ECM) proteins that are involved in tumor growth and metastasis, ECM deposition, and immunosuppressive microenvironment formation (19). CAFs modify tumor immunity by polarizing the adaptive immune response toward a protumor phenotype and influencing innate immune cell recruitment and activation (20). Specifically, they favor the tumor-promoting function of CD4^+^ T helper 2 lymphocytes over the tumor-protective T helper 1 response (21) and directly reduce the activation of CD8^+^ cytotoxic T cells and natural killer (NK) cells (22). CAFs also promote M2 macrophage polarization and induce the differentiation of Tregs to form the protumor microenvironment (23, 24). Therefore, understanding the effects of neoadjuvant therapy on CAFs is important for understanding the immune microenvironment.

Many markers have been used to identify and characterize CAFs, including α-smooth muscle actin (αSMA), fibroblast activation protein, fibroblast-specific protein 1, and podoplanin (PDPN) (25). αSMA is considered to be a unique marker of activated CAFs compared with normal fibroblasts (26). A study analyzed CAFs and stromal activation in neoadjuvantly treated and resected primary tumors using αSMA. The results showed that neoadjuvant therapy (gemcitabine/gemcitabine + erlotinib/gemcitabine + oxaliplatin/FOLFIRINOX/other regimens (such as radiotherapy)) may induce stromal depletion and reduce the number of CAFs (27, 28). Interestingly, tumors treated with the AG regimen had less CAF and stromal contents than those treated with conventional preoperative chemotherapy (gemcitabine + S-1), and the main effects were due to nab-paclitaxel and not gemcitabine (28, 29).

PDPN is a small cell-surface mucin-like glycoprotein that plays an important role in fibroblasts, macrophages, T helper cells, and epithelial cells and is associated with poor prognosis in several cancers. It acts as an endogenous ligand for the platelet C-type lectin receptor CLEC-2, which can elicit powerful platelet aggregation (30, 31). Platelets are rich in several growth factors, such as transforming growth factor-β, vascular endothelial growth factor-A, and CD40 ligand, which cause epithelial–mesenchymal transition, immune paralysis, and drug resistance (32). The neoadjuvant AG regimen resulted in a significant reduction in PDPN^+^ CAF contents and a decrease in extravasated platelet activation through PDPN^+^ CAF depletion, thereby preventing aggressive tumor cells from spreading, invading, and developing resistance to drugs (33).

CAFs have previously been considered to have tumor-promoting functions. However, recent studies targeting the hedgehog pathway have suggested that αSMA^+^ CAFs may have tumor-restraining functions (34, 35), which may be due to more in-depth research on the heterogeneity level of CAFs. Elyada et al. (36) applied scRNA-seq to pancreatic cancer tissue and found three distinct CAF subtypes: myofibroblastic CAFs (myCAFs), inflammatory CAFs (iCAFs), and antigen-presenting CAFs (apCAFs). myCAFs have elevated expression of αSMA, are closely adjacent to tumor cells, and are associated with ECM deposition (35, 37, 38), immunosuppression (39, 40), and tumor restraint (34, 35). iCAFs lack increased αSMA expression and instead secrete interleukin-6 (IL-6) and are distant from tumor cells (41). They are involved in immunosuppression and tumor promotion (39, 41) and may have overlapping functions with senescent fibroblasts (37). apCAFs have high expression of MHC II family genes and may perform antigen-processing and antigen-presentation functions (42). Notably, most current studies on neoadjuvant chemotherapy/chemoradiotherapy and CAFs are considered to involve all CAFs rather than specific CAF subtypes, as αSMA is regarded as a universal marker of activated CAFs. Due to the complex functional characteristics of CAF subtypes in pancreatic cancer, we emphasize the need to determine the relationship between neoadjuvant chemoradiotherapy and specific CAF subtypes to better inform the use of matrix-targeted therapy following or in combination with neoadjuvant chemoradiotherapy.

CAFs produce various extracellular matrix components, including fibronectin, laminin, hyaluronan, and collagens, of which type I, IV, or V collagens promote the malignant features of pancreatic cancer (43–45). Collagen has been reported to increase tumor tissue stiffness, regulate tumor immunity, and promote metastasis (46). After neoadjuvant therapy (FOLFIRINOX/AG/gemcitabine/gemcitabine + S1/S1 + irinotecan + oxaliplatin/S1 + radiation(50 Gy)/gemcitabine + S1 + radiation (50 Gy)), the expression of type I, III, IV, and V collagens in pancreatic cancer tissue was decreased (47). The mechanism of this remodeling may be that these neoadjuvant regimens reduced the expression of ephrin-A5, the upstream regulatory gene of collagen synthesis in CAFs, which leads to significant alterations in the expression level of the collagen gene, thus inducing a reduction in collagen volume in the TME (47). The remodeling of CAFs and collagens by neoadjuvant chemotherapy/chemoradiotherapy is associated with tumor softening and tumor shrinkage, which may allow the surgical removal of pancreatic cancer, but this softening is often difficult to detect on radiologic imaging.

The effect of neoadjuvant chemoradiotherapy on the level of interstitial fibrosis is controversial. As mentioned above, some studies suggest that some neoadjuvant chemotherapy or chemoradiotherapy regimens can reduce interstitial fibrosis by reducing CAFs. However, several studies seemed to support the concept that neoadjuvant chemoradiotherapy increases pancreatic cancer stromal fibrosis (48, 49), which may be secondary to tissue damage caused by preoperative chemoradiotherapy and subsequent reconstruction and repair. Two cell-surface proteins, a disintegrin and metalloprotease 10 and ephrinB2, are considered drivers of fibrosis, ECM, and epithelial–mesenchymal transition after neoadjuvant stereotactic body radiation therapy (49). In addition, the urokinase plasminogen activator activates plasminogen to form plasminase, which is involved in tissue degradation and proteolysis (50). Neoadjuvant FOLFIRINOX reduced PLAU expression levels (51), which may reduce the degradation of ECM structural components, increase fibrosis, and reduce tumor invasion and metastasis. Matsuda et al. (52) evaluated the pathological tissues of surgically resected specimens from patients undergoing neoadjuvant AG therapy and found that encapsulating mature fibrosis after neoadjuvant AG chemotherapy was associated with improved prognosis in patients with pancreatic cancer. This mature fibrosis refers to fine collagen fibers stratified into multiple layers with few or no fibroblasts. It forms a tumor bed at the same site where cancer cells accumulate before neoadjuvant therapy. The presence of mature fibrosis in the peri-tumor area may suggest that neoadjuvant AG chemotherapy may maintain effects for a longer time, thereby improving prognosis.

### 2.2 Tumor-associated macrophages

TAMs, an important type of infiltrating immune cells in the TME, are primarily divided into the antitumor M1 phenotype (classically activated state) and the protumor M2 phenotype (alternatively activated state), reflecting the Th1–Th2 polarization of T cells. M1 macrophages can directly mediate cytotoxicity or kill tumor cells through antibody-dependent cell-mediated cytotoxicity; M2 macrophages can inhibit the T cell-mediated antitumor immune response, promote tumor angiogenesis, and lead to tumor progression and metastasis (53). High M2 macrophage infiltration is known to be an independent predictor of poor survival (54).

Neoadjuvant therapy (gemcitabine/gemcitabine + erlotinib/gemcitabine + oxaliplatin/FOLFIRINOX/FOLFIRINOX + radiotherapy/other regimens (such as radiotherapy)) has been reported to drive the depletion of MDSCs and M2 macrophages (27, 54). Gemcitabine, an important agent in neoadjuvant therapy regimens, is thought to lower M2 macrophage mediators, including growth factors, such as platelet growth factors A and B and placental growth factor, and matrix proteins, such as osteopontin and fibronectin (55). In addition, gemcitabine-treated macrophages modulated the polarization of TAMs into an antitumor phenotype by increasing the expression of several immunostimulatory cytokines, such as IL-12, IL-27, and IFN-γ, as well as proinflammatory mediators, including prostaglandin-endoperoxide synthase, Fas ligand, and TNF-α (55). M1 and M2 macrophages are highly plastic, and their classification is influenced by therapeutic intervention or changes in the TME. Neoadjuvant low-dose irradiation has been reported to program the differentiation of iNOS^+^ M1 macrophages by inducing endothelial cell activation and Th1 chemokine expression and by inhibiting the production of angiogenic factors, immunosuppressive factors, and tumor growth factors (56). Other studies have shown that during the early stage after irradiation, macrophages polarize into the M1 phenotype, but in later stages, resident and recruited macrophages tend to differentiate into the M2 phenotype following vascular disruption and tumor hypoxia (57). Therefore, the effects of neoadjuvant radiotherapy on TAMs remain controversial and need further study.

Matsuki et al. (58) found that the number of M2 macrophages was significantly lower in female patients than in male patients after neoadjuvant chemoradiotherapy (gemcitabine + S-1 + 30-Gy radiation), which may be related to increased sex-dependent interferon regulatory factor 5 (IRF-5) expression in female patients. IRF-5 is a transcription factor that has been shown to have tumor suppression functions and plays a central role in regulating TAM polarization (59, 60). IRF-5 is thought to promote M1 macrophage polarization by equipping the cells with an IL-12^high^ IL-23^high^ IL-10^low^ cytokine profile and promoting Th1 and Th17 lymphocyte responses (61). Based on these findings, IRF-5 may be a key participant in the remodeling of the TME by neoadjuvant chemoradiotherapy, which warrants further study. In addition, the changes in immune cells after neoadjuvant chemoradiotherapy were related to their location in the TME. Okubo et al. (62) demonstrated that neoadjuvant chemoradiotherapy (S-1 + radiation) significantly reduced the M2 macrophage count in the nests of cancer cells, while little change was observed in the M2 macrophage count in the tumor stroma.

### 2.3 T cells

To evaluate the effect of neoadjuvant therapy on the immune microenvironment of pancreatic cancer, many researchers have examined resected specimens from patients who received neoadjuvant chemoradiotherapy and those who did not receive neoadjuvant therapy with immunohistochemical staining or flow cytometry. Homma et al. (63) found that the number of CD4^+^ and CD8^+^ lymphocytes in patients receiving neoadjuvant gemcitabine plus S-1 followed by radiation therapy was significantly higher than that in patients not receiving neoadjuvant therapy. Tsuchikawa et al. (64) found that neoadjuvant gemcitabine-based chemotherapy with or without chemoradiotherapy reduced the number of Foxp3^+^ Tregs but did not induce CD4^+^ or CD8^+^ lymphocyte infiltration. Shibuya and others suggested that the number of myeloid cells and Tregs in the pancreatic cancer microenvironment was significantly reduced after neoadjuvant chemotherapy (gemcitabine + docetaxel + capecitabine) followed by chemoradiotherapy. Although the number of CD8^+^ cells was also significantly decreased, the ratio of CD4^+^ and CD8^+^ cells to Tregs was significantly higher (65). Michelakos et al. (54) found that neoadjuvant FOLFIRINOX with or without chemoradiotherapy reduced human leukocyte antigen (HLA)-A defects, increased CD8^+^ cell density, and decreased Treg density. Mota Reyes et al. (27) found an effect of neoadjuvant therapy (gemcitabine/gemcitabine + erlotinib/gemcitabine + oxaliplatin/FOLFIRINOX/other regimens (such as radiotherapy)) on enriching antitumor immune cells in the TME, especially the selective deletion of Tregs and MDSCs, which is related to the increase in T-cell antitumor activity. Okubo and his colleagues showed that neoadjuvant S‐1 plus radiation therapy reduced the overall immune cell count, but these changes were heterogeneous in the cancer cell nests and cancer stroma. The number of CD4^+^ T cells, CD20^+^ B cells, and Foxp3^+^ T cells in the cancer stroma was significantly reduced in patients who received neoadjuvant S‐1 plus radiation therapy compared to those who did not, whereas the count of these cells in the cancer cell nests did not significantly differ between groups (62).

The effect of neoadjuvant chemoradiotherapy on T cells is complex and important. The depletion of Tregs and the increase in the CD8^+^ T/Treg ratio by the aforementioned neoadjuvant regimen would improve the survival benefit. Low Treg frequency, high CD8^+^ T-cell frequency, high CD4^+^ T-cell frequency, and high CD8^+^ T/Treg ratio can be used as predictors for pancreatic cancer patients treated with neoadjuvant chemotherapy (66, 67). Targeting the TME to increase CD4^+^ and CD8^+^ T-cell counts and the CD8^+^/FOXP3^+^ cell ratio could decrease tumor recurrence and improve clinical outcomes (68). In addition, the effects of neoadjuvant chemotherapy on CD4^+^ and CD8^+^ T cells differed among these studies, which may be related to drug types, treatment cycles, detection methods, and heterogeneity of the patient population. Notably, T-cell subtypes have different functional characteristics, and thus, more unique markers need to be tested to further determine the effect of neoadjuvant chemotherapy on different T-cell subsets. In summary, clinical responders to neoadjuvant therapy may present an immunologic landscape of increased antitumor T lymphocytes and decreased Tregs, the mechanisms of which are discussed below.

#### 2.3.1 Neoadjuvant therapy activates T-cell effector molecules

Neoadjuvant FOLFIRINOX has been found to increase the expression of CD44 (a T-cell activation marker) and granzyme B (a protease related to the killing function of cytotoxic T cells), reflecting increased CD8^+^ T-cell activation (69). Patients who received the neoadjuvant AG and FOLFIRINOX regimens exhibited a strong tendency to show decreased expression of programmed death-ligand 1 (PD-L1) and CD47 on tumor cells, which restored the attack of T cells and macrophages on tumors, respectively (70). After neoadjuvant stereotactic body radiation and IL-12 combination therapy, intratumoral interferon-γ production increased, which initiated inhibitory cell reprogramming and subsequently increased CD8^+^ T-cell activation (71). Furthermore, gemcitabine enhanced the killing function of cytotoxic T lymphocytes by upregulating Fas expression on pancreatic cancer cells (72). Paclitaxel not only directly activated effector T cells by raising the production of the Th1 cytokines interferon-γ and IL-2 and by increasing the expression of the activator marker CD44 in CD4^+^ and CD8^+^ T cells but also indirectly reduced the inhibitory function of Tregs by upregulating CD95 (73, 74). Delitto et al. (75) found higher levels of IL-2 in fresh pancreatic cancer surgical specimens after neoadjuvant gemcitabine-based chemotherapy, which was associated with T-cell proliferation and activation.

#### 2.3.2 Neoadjuvant therapy regulates T-cell infiltration

High interstitial pressure and vascular collapse of the pancreatic TME limit T-cell entry to the tumor sites (76). Abnormal vasculature reduces oxygen supply, resulting in hypoxia in the TME. Hypoxia promotes CAF recruitment, connective tissue hyperplasia and activation (77), and transforming growth factor-β expression in CAFs, resulting in the exclusion of CD8^+^ T cells from the tumor parenchyma to hinder antitumor immunity following immunotherapy (78, 79). Hypoxia also facilitates the recruitment of MDSCs and inhibits the activity of T lymphocytes (80). The reduction in CAFs and interstitial activation induced by the above neoadjuvant regimens may facilitate T-cell infiltration into the tumor sites. Neoadjuvant local low-dose γ irradiation normalizes the aberrant vasculature and induces iNOS expression in TAMs, thereby enhancing tumor-specific T-cell recruitment and T cell-mediated tumor rejection in pancreatic cancer (56). Chemotherapy has been reported to induce intratumoral chemokine expression, favoring T-cell infiltration (81). Because T-cell infiltration in pancreatic cancer is directly correlated with a distinct panel of four chemokines, CCL4, CCL5, CXCL9, and CXCL10 (82), we hypothesize that neoadjuvant therapy increases T-cell infiltration by influencing these key chemokines, which needs to be confirmed by more basic experiments.

#### 2.3.3 Neoadjuvant therapy promotes antigen presentation

Dying cancer cells release damage-associated molecular patterns (DAMPs), such as calreticulin, heat shock protein (HSP), and high mobility group box protein 1, into the TME, which promote the recruitment and activation of antigen-presenting cells (APCs) by acting on different pattern recognition receptors (83, 84). Mature APCs can phagocytize antigenic material, migrate to lymph nodes, present antigens on MHC molecules to T cells, and trigger a T-cell immune response (85). Considered “eat me signals”, Hsp70 and calreticulin can interact with a variety of APC surface receptors, such as CD91 and CD40, to promote the cross-presentation of antigens on MHC class I molecules, thus triggering a tumor-specific CD8^+^ T-cell response (86–88). MHC class I-related chains A and B (MICA/B), as a component of DAMPs, enhance the cytolytic responses of NK cells, γδ T cells, and CD8^+^ αβ T cells by binding to natural killer group 2 member D (89, 90).

Neoadjuvant chemoradiotherapy (gemcitabine + S-1 followed by radiation therapy (30 Gy)) has been reported to induce the overexpression of DAMPs (such as MICA/B, calreticulin, and Hsp70), increase the number of CD8^+^ and CD4^+^ tumor-infiltrating T lymphocytes, and decrease the ratio of Treg/tumor-infiltrating T lymphocytes (90). This finding implies that this neoadjuvant chemoradiotherapy may stimulate T-cell immune responses by promoting antigen presentation through DAMPs (such as calreticulin and Hsp70) or directly enhance the cytolytic responses of CD8^+^ αβ T cells, NK cells, and γδ T cells by inducing the overexpression of MICA/B. In addition, radiation can also enhance antigen processing and calreticulin exposure, resulting in enhanced T-cell killing (91).

#### 2.3.4 Neoadjuvant therapy depletes tumorigenic immune cells and induces neoantigen release

Neoadjuvant chemotherapy (such as FOLFIRINOX and some gemcitabine-based regimens) aims to increase the accumulation of tumor-specific T cells in the peritumoral niche by selectively depleting protumorigenic immune cells (27, 65). Treg ablation restores immunogenic tumor-associated CD11c^+^ dendritic cells (DCs) and activates CD8^+^ T cells (92). MDSC depletion may also be a mechanism of neoadjuvant chemotherapy-induced increase in T cells because MDSCs can control T-cell responses by inducible nitric oxide synthase and arginase 1 (93).

Mutation-associated neoantigens have been shown to be the target of powerful antitumor T-cell responses. Due to the presence of these neoantigens, patients can mount endogenous immune responses, and the antigen-specific T cells are expanded (94). Neoadjuvant chemoradiotherapy induces new mutations in rectal cancer, resulting in the generation of neoantigens and changing the immune function of patients (95). Radiotherapy is thought to increase the expression of genes encoding immunogenic mutated proteins that are presented by MHC-I and MHC-II molecules, thereby triggering CD8^+^ and CD4^+^ T-cell responses (96). Therefore, we predict that the T-cell response promoted by neoadjuvant chemoradiotherapy may be related to the expression of strong neoantigens in pancreatic cancer.

### 2.4 B cells and T follicular helper cells

B lymphocytes play a dual role in tumor immunity. As a promotive effect, B cells produce antibodies to participate in humoral immunity and activate T cells to participate in cellular immunity. Alternatively, IL-10 from B cells inhibits cytotoxic T-cell activation to play a critical role in immunosuppression (97). The number and function of CD19^+^CD20^+^ B cells decreased in response to neoadjuvant therapy (gemcitabine/gemcitabine + erlotinib/gemcitabine + oxaliplatin/FOLFIRINOX/S-1/other regimens (such as radiotherapy)) (27, 69, 98). Notably, MZB1 (marginal zone B- and B1-cell-specific protein) was detected in the tumor stroma after neoadjuvant chemoradiotherapy and was positively correlated with elevated accumulation of CD8^+^ T lymphocytes (99). MZB1 upregulated tumor-specific antibody secretion and decreased IL-10 expression by regulating the storage of calcium ions in the endoplasmic reticulum, which activated antitumor immunity (99). According to our previous study, T follicular helper cells (Tfhs) recruited CD8^+^ T cells and B cells by secreting CXCL13 and promoted the maturation of B cells into antibody-producing plasma cells by secreting IL-21, thereby promoting the formation of an immunostimulatory TME (100). The function of Tfhs was inhibited by the PD-L1/programmed cell death protein 1 (PD-1) signaling pathway in pancreatic cancer, which was reversed after neoadjuvant AG chemotherapy (100).

### 2.5 Other immune cells and tertiary lymphoid organs

DCs, considered the most effective APCs in tumor immunity, have exhibited marked expansion after neoadjuvant therapy (gemcitabine/gemcitabine + erlotinib/gemcitabine + oxaliplatin/FOLFIRINOX/other regimens (such as radiotherapy)) (27). As mentioned earlier, neoadjuvant chemoradiotherapy (gemcitabine + S-1 followed by radiation therapy (30 Gy)) triggered the release of immunostimulatory DAMPs, which activated DCs through toll-like receptor 4 (101). Gemcitabine-based neoadjuvant therapy was found to increase FMS-like tyrosine kinase 3 ligand (Flt3L) concentrations (75). Flt3L is a growth factor, and the main effect of the Flt3/Flt3L axis is to regulate DC generation, development, maturation, expansion, and homeostasis (102, 103). Flt3L might synergize successfully with other therapies that promote tumor antigen availability by increasing the number of DCs.

Intratumoral NK-cell infiltration constitutes an independent prognostic factor for pancreatic cancer (27). After neoadjuvant therapy (gemcitabine/gemcitabine + erlotinib/gemcitabine + oxaliplatin/FOLFIRINOX/S-1/other regimens (such as radiotherapy)), the number of CD56^+^ NK cells was significantly reduced (27), and their functions were notably decreased (98). The mechanism of the alterations in NK cells after neoadjuvant therapy is unclear. However, studies have shown that neoadjuvant chemoradiotherapy (gemcitabine + S-1 followed by radiation therapy (30 Gy)) induces the overexpression of MHC class I-related chain A/B (90), a membrane protein expressed in cancer cells that is induced by irradiation or chemotherapy, and interacts with natural killer group 2 member D to enhance the killing effect of NK cells on cancer cells (89, 104, 105).

Neoadjuvant FOLFIRINOX therapy prevents tumor-associated neutrophil (TAN) accumulation by affecting the CXCL5–CXCR2 axis and decreasing intratumoral IL-8 levels (51). CXCL5 is a chemokine for neutrophils, and CXCR2 is essential for the recruitment of TANs (106). Similarly, IL-8 can elicit massive neutrophil accumulation and activation (107). In the absence of neutrophils, activated and functional T cells infiltrate pancreatic tumors otherwise devoid of effector T cells (108). However, few reports have examined the effects of neoadjuvant therapy on eosinophils, basophils, or mast cells.

Tertiary lymphoid organs (TLOs) are accumulations of lymphoid cells that recognize antigens and produce antigen-specific antibodies in the immune system (109). The proportion of CD8^+^ T lymphocytes, PNAd^+^ high endothelial venules, CD163^+^ macrophages, and Ki‐67^+^ cells within the TLO was higher after neoadjuvant chemotherapy (gemcitabine/S-1/gemcitabine + S-1/FOLFIRINOX) with/without radiation, while the proportion of PD-1^+^ immunosuppressive lymphocytes within the TLO was lower (110). By inducing T-cell infiltration and the development of TLO in the TME, “nonimmunogenic” tumors can be transformed into “immunogenic” tumors (111).

## 3 More promising treatment strategies

**Figure 2:**
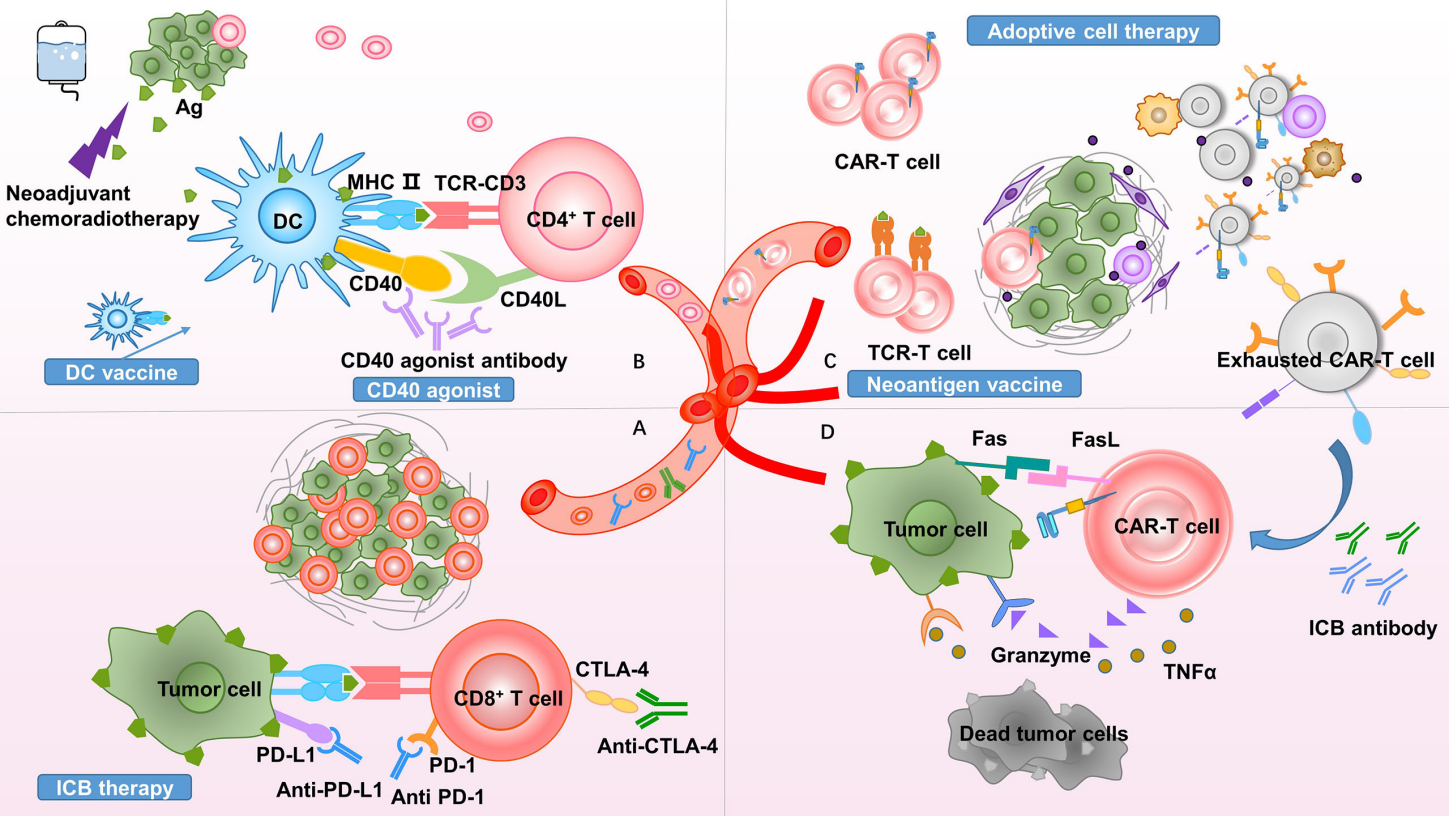
Theoretical basis of neoadjuvant combination therapy. Proposed rationales to clarify the promise of neoadjuvant combination therapy. **(A)** Neoadjuvant therapy increases immunotherapy targets by promoting effector T-cell infiltration and increasing ICB antibody delivery. **(B)** Due to the presence of the tumor burden serving as an antigen source, neoadjuvant therapy promotes antigen presentation and improves responses to CD40 agonists. **(C)** Neoadjuvant therapy increases the infiltration of CAR-T cells and TCR-T cells into the tumor site. The inhibitory TME exhausts T cells, upregulating inhibitory molecular receptors, such as PD-1, TIM-3, and LAG-3. **(D)** ICB reverses T-cell exhaustion and restores antitumor immunity. PD-1, programmed cell death protein 1; PD-L1, programmed death-ligand 1; CTLA-4, cytotoxic T-lymphocyte-associated antigen 4; MHC, major histocompatibility complex; Ag, antigen; DC, dendritic cell; TCR, T-cell receptor; CAR, chimeric antigen receptor; TIM-3, T-cell immunoglobulin and mucin-domain containing-3; LAG-3, lymphocyte-activation gene 3; ICB, immune checkpoint blockade; TNF, tumor necrosis factor; TME, tumor microenvironment.

In summary, neoadjuvant therapy alters the pancreatic cancer microenvironment by reducing CAFs and interstitial fibrosis; decreasing the infiltration of immunosuppressive cells, including M2 macrophages, MDSCs, TANs, and Tregs; and increasing cytotoxic T-cell recruitment. This change creates favorable conditions for immunotherapy. We list some clinical trials of neoadjuvant chemotherapy or chemoradiotherapy combined with immunotherapy (**Table 1**). We speculate that neoadjuvant chemoradiotherapy combined with immunotherapy is promising, and this strategy is discussed below (**Figure 2**). Notably, the efficacy of neoadjuvant combination strategies needs to be tested in more clinical trials.

**Table 1:** Clinical trials of neoadjuvant chemotherapy or chemoradiotherapy combined with immunotherapy.

NCT number	Neoadjuvant interventions	Phase	Status
NCT05132504	FOLFIRINOX + pembrolizumab	1	Not yet recruiting
NCT03970252	FOLFIRINOX + nivolumab	1	Recruiting
NCT02548169	FOLFIRINOX + gemcitabine + nab-paclitaxel + DC vaccine	1	Terminated
NCT04940286	Gemcitabine + nab-paclitaxel + durvalumab + oleclumab	2	Recruiting
NCT02588443	Gemcitabine + nab-paclitaxel + RO70097890	1	Completed
NCT02030860	Gemcitabine + nab-paclitaxel + paricalcitol	Not applicable	Completed
NCT02405585	mFOLFIRINOX + SBRT + algenpantucel-L	2	Terminated
NCT02451982	Cyclophosphamide + nivolumab + GVAX pancreas vaccine + SBRT	2	Active, not recruiting

FOLFIRINOX, folinic acid + irinotecan + oxaliplatin + leucovorin; DC, dendritic cell; SBRT, stereotactic body radiation therapy; NCT, National Clinical Trial.

GVAX pancreas vaccine: a granulocyte-macrophage colony-stimulating factor-modified whole-cell tumor vaccine. Not yet recruiting: the study has not started recruiting participants. Active, not recruiting: the study is ongoing, and participants are receiving an intervention or being examined, but potential participants are not currently being recruited or enrolled.

### 3.1 Immune checkpoint blockade therapy

Currently known immunotherapy targets focus on cytotoxic T cells, including PD-1, cytotoxic T-lymphocyte-associated antigen 4 (CTLA-4), T-cell immunoglobulin and mucin-domain containing-3 (TIM-3), lymphocyte-activation gene 3 (LAG-3), and T-cell immunoglobulin and ITIM domain (TIGIT). The premise of effective immunotherapy is efficient T-cell infiltration, and the level of T-cell infiltration at baseline is of great significance for the prediction and prognosis of immune checkpoint blockade (ICB) (112, 113). Although activated T cells are observed on the periphery of tumors, they will not respond to ICB if they cannot infiltrate the tumor itself (114). Neoadjuvant chemoradiotherapy may increase T-cell infiltration into the TME by increasing tumor antigen release and normalizing vasculature, providing targets for ICB. Chemotherapy combined with immunotherapy is predicted to reinforce and synergistically increase the antitumor effects of either therapy alone (115). A phase Ib study of pembrolizumab, an anti-PD-1 antibody, combined with chemotherapy enrolled 49 patients with advanced metastatic solid tumors, including 11 pancreatic cancer patients. The results showed that the clinical response rate was approximately 92%, and standard doses of pembrolizumab could be safe in combination with gemcitabine and nab-paclitaxel (116). Radiotherapy also provides targets for immunotherapy by regulating the expression of vascular endothelial cell adhesion molecules and chemokines to enhance lymphocyte infiltration (117). Many clinical trials concerning ICB therapy combined with neoadjuvant therapy in pancreatic cancer are recruiting or are about to begin recruiting patients (NCT02305186 and NCT05132504), and their results are to be expected. In addition, the antistromal effect of neoadjuvant chemoradiotherapy may increase ICB antibody delivery, enabling ICB-resistant patients to respond again.

### 3.2 CD40 agonist therapy

CD40 is a member of the tumor necrosis factor receptor superfamily expressed on the surface of various immune cells, including APCs, and in some tumor cells (118). CD40 agonists enable DCs to increase the levels of critical T-cell stimulatory cytokines, including IL-12, which potentiates T-cell activation (119). The addition of chemotherapy prior to administering agonist CD40 monoclonal antibody (mAb) has been shown to prime APCs by releasing tumor-associated antigens through cytotoxic cell death, improving responses to CD40 agonists (120). To test this hypothesis, a phase I trial combining an agonist CD40 mAb (CP-870,893) with gemcitabine was conducted on patients with advanced pancreatic cancer (121). The combination treatment was well tolerated and associated with objective tumor responses in 22 (19%) patients (121). In another phase 1b study, the CD40 agonistic mAb APX005M (sotigalimab) was combined with gemcitabine and nab-paclitaxel, with or without nivolumab, in patients with metastatic pancreatic ductal adenocarcinoma (122). Combination therapy achieved a 58% response rate among 24 patients (122).

Moreover, CD40 mAb synergizes with chemotherapy and radiotherapy to sensitize tumors that had previously resisted treatment with anti-CTLA-4 or anti-PD-1/PD-L1 mAb, further stimulating tumor regression and improving survival (123, 124). Byrne et al. (125) conducted a phase 1 trial and demonstrated that the neoadjuvant agonist CD40 mAb (selicrelumab) with or without chemotherapy can modulate the TME in pancreatic cancer, including reducing tumor stromal density, activating DCs, re-educating macrophages, and increasing T-cell infiltration. In addition, they found that the expression of LAG-3, PD-1, and TIGIT was significantly increased on CD4^+^ and CD8^+^ T cells in the TME, implying that combined ICB therapy may provide greater clinical synergistic benefit.

### 3.3 Adoptive cell therapy

Adoptive cell therapy is a type of immunotherapy for eliminating cancer by directly delivering *in vitro* activated and expanded tumor-specific or non-specific killer cells to patients. In addition to the well-known T cells (126), γδT cells (127), NK cells (128), NKT cells (129), and even macrophages (130) are engineered to express antigen-specific T-cell receptors or chimeric antigen receptors (CARs) (131). To date, CAR-T-cell therapy has achieved success mainly in hematological malignancies. Despite extensive research, CAR-T-cell therapy has not proven effective in solid tumors (132). The trafficking and infiltration of CAR-T cells into tumor cells limit the effectiveness of CAR-T-cell therapy (133). As mentioned above, the number of T cells in the TME of some patients increased after neoadjuvant chemotherapy, and these increased T cells may come from tissue-resident T cells or may be recruited from the bone marrow or periphery, which reflects that the TME after neoadjuvant therapy may be conducive to the existence of T cells. In addition, the reduction in CAFs after neoadjuvant therapy may break down the dense mechanical barrier of pancreatic cancer and promote T-cell infiltration into the tumor site, which may improve the function of CAR T cells. However, once CAR-T cells enter the tumor, sustained antigen exposure, suppressive immune cells, and suppressive cytokines in the TME cause the function of T cells to gradually degenerate (134). This condition, known as “exhaustion”, is characterized by attenuated effector cytotoxicity, reduced cytokine production, and increased expression of multiple inhibitory molecular receptors, such as PD-1, TIM-3, and LAG-3 (135). Exhausted CAR-T cells with impaired proliferation and persistence cannot efficiently kill malignant clones, usually leading to treatment failure (133). The reduction in MDSCs and Tregs after neoadjuvant therapy attenuates the immunosuppressive state of the TME, creating an improved TME to alleviate CAR-T exhaustion. In addition, targeting immune checkpoint pathways (such as PD-1 and CTLA-4) can relieve the exhaustion of CD8^+^ T cells and renew their priming (136, 137). Combining CAR-T-cell therapy with ICB or depleting other suppressive factors in the TME has shown very promising results for mitigating the phenomenon of T-cell exhaustion (132).

### 3.4 Dendritic cell therapy

DCs enhance the immune response by presenting antigens to T cells. Based on their powerful antigen presentation capability, DCs are designed as vaccines loaded with tumor antigens to promote antigen-specific antitumor T-cell immunity. Recent studies suggest that DC vaccines in combination with chemotherapy or ICB or CD40 agonists may be a promising therapeutic approach. Reducing tumors by neoadjuvant chemotherapy is considered to improve DC therapy because DC therapy is most effective in the case of low tumor burden (138). In addition, the depletion of suppressive immune cells, including Tregs and MDSCs, also facilitates the synergistic effect of neoadjuvant chemoradiotherapy combined with DC therapy. Promoting tumor-specific immunity with DC vaccines and then amplifying it with ICB is an attractive direction. Clinical evidence suggests that the combination of DC therapy and ICB is more effective than using DC therapy as monotherapy (139, 140). The blockade of PD-L1 on DCs promotes the activation of T cells by DCs, downregulates IL-10 secretion by Th2 cells, and upregulates IL-2 secretion by Th1 cells (141). Lau et al. (142) investigated the efficacy of a DC vaccine in combination with CD40 agonism in a mouse model of pancreatic cancer with poor immunogenicity. The results showed that although CD40 agonist monotherapy ameliorated intratumoral T-cell infiltration, these cells showed marked exhaustion. CD40 agonists combined with DC vaccines can improve T-cell exhaustion and reduce tumor progression.

### 3.5 Neoantigen vaccination

Neoantigens are somatic mutations expressed only by tumor cells (143). Because of their specific expression in tumors, neoantigens can only destroy tumor cells without causing excessive damage to important healthy tissues (144). Therefore, neoantigens are ideal targets for cancer immunotherapy. DCs loaded with neoantigens have been reported to trigger specific T-cell responses in melanoma patients (145). Another approach to target neoantigens is to transfer engineered T cells expressing the tumor-specific T-cell receptor into the host. In a recent study, a patient with progressive metastatic pancreatic cancer was treated with autologous T cells that had been genetically engineered to clonally express two allogeneic HLA-C*08:02-restricted T-cell receptors (TCRs) targeting mutant KRAS G12D expressed by the tumors. Visceral metastases regressed in this patient, and the response was ongoing at 6 months (146). Since most neoantigens are derived from patient-specific mutations, neoantigen T-cell receptor gene therapy is highly personalized (147). In conclusion, we believe that cancer immunotherapy targeting neoantigens is a promising treatment option, but methods to reduce the cost of this highly personalized treatment warrant more research.

## 4 Conclusions

The beneficial effects of neoadjuvant chemotherapy do not depend on the direct cytotoxicity of chemotherapeutic agents, as traditionally thought. Instead, these effects may be mediated by the restoration of antitumor responses *via* alterations in the phenotype of the immune microenvironment. These alterations are caused by remodeling antigen exposure and the distribution of immune cells in the TME. This review summarizes the changes and possible mechanisms of the pancreatic cancer immune microenvironment in response to neoadjuvant therapy. We found that many neoadjuvant regimens (such as AG or FOLFIRINOX) led to an immunological shift toward a more effective antitumor immune response in the TME, providing an impetus for the possibility of combining neoadjuvant chemotherapy or chemoradiotherapy with immunotherapy and highlighting the urgent need for combined neoadjuvant therapeutic strategies. Theoretically, the impact of neoadjuvant therapy on the immune microenvironment is heterogeneous in the population. Some patient groups will follow the direction of promoting inflammation and anticancer activity, while others will not. The selection of subsets of patients who can benefit from neoadjuvant therapy remains a problem to be solved. We suggest that the short time between cancer diagnostic biopsy and primary surgery can be used to screen for neoadjuvant therapies that induce a response-associated TME. Current studies have not been able to include the changes of all components in the TME, and we look forward to more discoveries in this area to develop more effective treatments to eradicate the disease in the future.

## Author contributions

All authors contributed to the article and approved the submitted version.

## Funding

This study was jointly supported by the National Natural Science Foundation of China (U21A20374, 82173091, and 81701630), Shanghai Municipal Science and Technology Major Project (21JC1401500), Scientific Innovation Project of Shanghai Education Committee (2019-01-07-00-07-E00057), Clinical Research Plan of Shanghai Hospital Development Center (SHDC2020CR1006A), Xuhui District Artificial Intelligence Medical Hospital Cooperation Project (2021-011), Shanghai Natural Science Foundation (22ZR1412900), Research Project of Shanghai Municipal Health Commission (20214Y0396, 20194Y0375), and Shanghai Pujiang Program (2021PJD014). The funding agencies had no role in study design, data collection and analysis, decision to publish, or preparation of the manuscript.

## Conflict of interest

The authors declare that the research was conducted in the absence of any commercial or financial relationships that could be construed as a potential conflict of interest.

## Publisher’s note

All claims expressed in this article are solely those of the authors and do not necessarily represent those of their affiliated organizations, or those of the publisher, the editors and the reviewers. Any product that may be evaluated in this article, or claim that may be made by its manufacturer, is not guaranteed or endorsed by the publisher.

## Supplementary material

Supplementary Figure 1 The flowchart of searching strategies.
