# Supplementary figures and images for: Neoadjuvant therapy alters the immune microenvironment in pancreatic cancer
Source: Front Immunol. 2022 Sep 26;13:956984. doi: 10.3389/fimmu.2022.956984 (PMC9548645; doi:10.3389/fimmu.2022.956984)

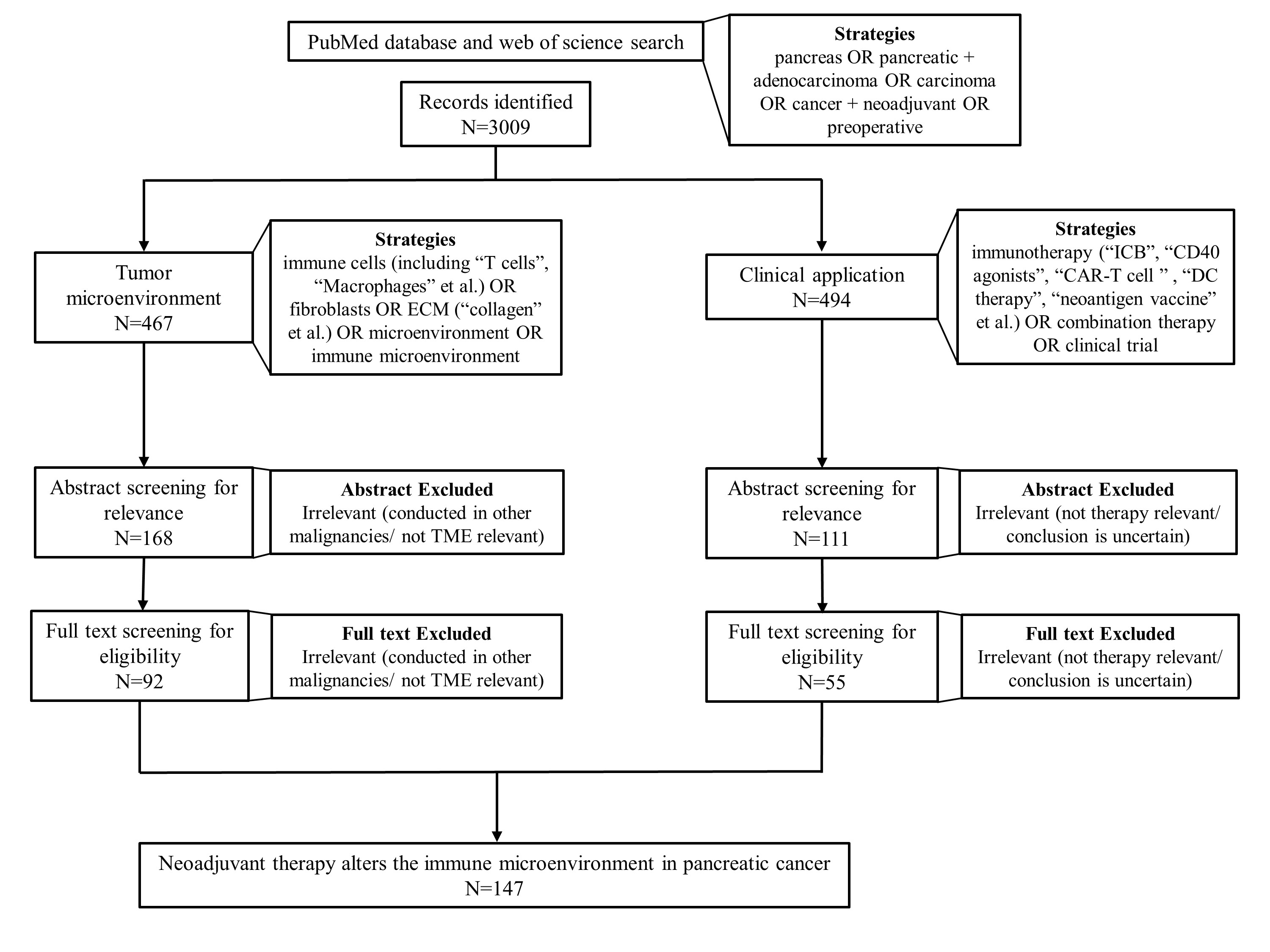

Supplement: Supplementary Figure 1 — The flowchart of searching strategies. [file Image_1.jpeg]
